# Thymic hyperplasia in a patient with Grave's disease

**DOI:** 10.1186/1755-7682-5-6

**Published:** 2012-02-09

**Authors:** Amira A Hamzaoui, Rim R Klii, Randa R Salem, Ines I Kochtali, Mondher M Golli, Silvia S Mahjoub

**Affiliations:** 1 Department of Internal Medicine and Endocrinology- Fattouma Bourguiba Hospital- Monastir 5000- Tunisia; 2 Department of Radiology- Fattouma Bourguiba Hospital- Monastir 5000- Tunisia

**Keywords:** Grave's disease, thymic hyperplasia, Hyperthyroidism

## Abstract

Hyperplastic changes of the thymus may be found in patients with Graves' disease. However, this rarely presents as an anterior mediastinal mass, particularly among adults. In this report, we describe a 46-year old woman with Graves' disease and thymic hyperplasia.

## Introduction

Thymic hyperplasia (TH) is a common feature in Graves' disease (GD) and the relationship between GD and thymic changes is discussed [1,2]. However, in most cases, thymic enlargement is minimal. Recognizing the association between TH and GD, and existence of the benign course after treatment of the hyperthyroidism may be useful for avoiding unnecessary surgical procedure.

We report a 46-year-old 40 × 30 × 50 mm, woman with GD and hyperthyroidism. She had an anterior mediastinal mass that was diagnosed as TH and disappeared after treatment of the hyperthyroid state.

## Case report

A 46 year-old-woman was referred to our hospital, because of weakness and hemopthaesia. Medical history, social history, and family history were non contributory, and the patient took no medications. Her physical examination was normal. The thyroid gland did not appear prominent. Results of routine laboratory studies were all normal. Thyroid function tests demonstrated as follows: free T4 to be 5.8 ng/dL (normal range, 1.0 to 1.8), and thyroid-stimulating hormone to be < 0.005 u IU/mL (normal range, 0.3 to 4.0). Comptud tomography scan of the chest revealed well-circumscribed soft tissue density mass, 30 mm in size, with a regular periphery in the anterior médiastinum.

Thyroid-directed antibodies were negatives: anti-thyroglobulin antibody, and antimicrosomal antibody. TSH-receptor antibody were positive. Anti-acetylcholine receptor antibodies and prostigmine test were negatives.

The diagnosis of Grave's disease associated to thymoma was made. She was treated with 30 mg per day of Benzylthiouracile (200 mg per day) for several weeks and became clinically euthyroid. Three months later surgery, the anterior mediastinal mass disappeared on a repeat computed tomographic scan of the chest.

## Discussion

Thymic hyperplasia is a common and reversible feature in patients with GD and hyperthyroidism [1,2]. In most cases, thymic hypertrophy is minimal and unapparent. Therefore, radiologically detectable thymic enlargement as an anterior mediastinal mass with thyrotoxicosis has been infrequently reported. Half of these cases undergo thymectomy because they are suspected of having thymoma. Recognition of the benign nature of TH and its regression following treatment of the hyperthyroidism is important to prevent unnecessary surgical procedures

Michie and Gunn report that approximately 38% of patients with thyrotoxicosis have histologic changes of the thymus gland [6].

Graves disease is an autoimmune disorder characterized by thyroid enlargement and hyperthyroidism. Thyrotropin receptor (TSHR) autoantibodies bind the TSHR on the membrane of thyroid follicular cells and stimulate cell proliferation and thyroid-hormone synthesis. TSHR has also been identified in extrathyroidal organs, including the human thymus [7].

Inoue and all conclude that anterior mediastinal mass (AMM) in GD could thus sometimes turn out to be TH and not a thymoma [8]; however, Levy and Lee noted that GD can also be associated with invasive malignant thymoma [9,10].

TH is a common feature in GD. However, in most cases, thymic enlargement is minimal, and radiologically detectable massive enlargement of the thymus is infrequently reported. Half of them undergo thymectomy due to the concern about a thymoma [11].

The differential diagnosis of an AMM includes several malignant lesions with a risk often warranting early surgical excision. In light of the association of benign TH with GD, thymectomy may be delayed in expectation of thymic regression with medical therapy. The timing of regression is variable, and very few reports exist in the literature [1-12].

The mechanism of TH in hyperthyroidism and GD is not well established. Van Herle and Chopra [13] described that hyperthyroidism persists after thymectomy, and Scheiff and colleagues [14] reported that administration of triiodothyronine can induce thymic enlargement in mice. Murakami and colleagues [15] investigated thymic size and density in 23 untreated patients with GD and 38 control subjects using computed tomography. The patients with GD had larger thymic size and higher thymic density than age-matched control subjects. After treatment with anti-thyroid drugs, both thymic size and density were significantly reduced with a concomitant decrease in thyroid-stimulating hormone receptor antibodies. Murakami and colleagues [15] also clearly showed the presence of thyrotropin receptors in the nonneoplastic thymic tissue by polymerase chain reaction amplification, Northern and Western blot analysis, and immunohistochemistry. These results indicate that TH is apparently associated with GD, and suggest that a thymic thyrotropin receptor may act as an autoantigen that may be involved in the pathophysiology of development of GD.

## Conclusion

If an AMM in a thyrotoxic patient is detected on a computed tomographic scan of the chest, and if it is a homogeneous mass with no invasion to the neighboring tissue, and no calcification, no septum, and no cystic lesion, then a high priority should be given to the treatment of the patient's hyperthyroidism under close radiologic follow-up of the AMM. If the size of the mass does not decrease in spite of keeping a euthyroid state for several months, we should take minimal invasive diagnostic maneuvers such as a thoracoscopic procedure or a cervical approach.

## Competing interests

The authors declare that they have no competing interests.

## Authors' contributions

AH: Wrote the manuscript; RK participate to the writing of the manuscript; RS: Done the radiologic exploration; IK: participate to the writing of the manuscript; MG: Done the radiologic exploration; SM: Participate to the coordination. All authors read and approved the final manuscript.

## Consent statement

'Written informed consent was obtained from the patient for publication of this case report. A copy of the written consent is available for review by the Editor-in-Chief of this journal.'
